# Documenting Behavioral Health Needs in an Urban Setting

**DOI:** 10.3389/fpubh.2021.493884

**Published:** 2021-08-30

**Authors:** Darrell Hudson, Stacey McCrary, Vithya Murugan, Lara Gerassi, Enola K. Proctor

**Affiliations:** ^1^Brown School at Washington University in St. Louis, St. Louis, MI, United States; ^2^College for Public Health and Social Justice, Saint Louis University, St. Louis, MI, United States; ^3^School of Social Work, University of Wisconsin-Madison, Madison, WI, United States

**Keywords:** urban, needs assessment, behavioral health, mixed method approach, race/ethnic

## Abstract

Most local communities lack the capacity to conduct behavioral health needs assessments. The purpose of this paper is to describe a mixed-methods approach to estimate the behavioral health needs in St. Louis, MO. Data were drawn from multiple sources including local and state government prevalence estimates, medical records, and key informant interviews. The most prevalent behavioral conditions were depression, alcohol, and drug abuse. Priority populations were residents with co-occurring disorders, youth transitioning into the adult behavioral system, and homeless individuals with behavioral health needs. Treatment rates for behavioral health conditions were low, relative to identified needs. There are significant provider shortages and high staff turnover, which extend wait times, diminish the quality of care, and contribute to the use of emergency departments for behavioral health care. The data and methods described in this paper could be helpful to other municipalities that are looking to conduct behavioral health needs assessments.

## Introduction

Behavioral health conditions comprise the leading source of disability in the U.S., accounting for nearly 40% of all medical disability for those age 15–44 (1, 2). This disability burden underscores the importance of behavioral health services addressing not only symptom management but also outcomes of social functioning and well-being (1, 3). For example, it is estimated that over a 2-week period, 8% of the U.S. adult population aged 20 or older has depression (4).

Although rates of behavioral health treatment have increased over time, lack of access, and quality remain serious problems (5–8). For example, fewer than half of people with major depressive disorder receive depression care and among those who do receive treatment, only about 20% receive minimally adequate care (9). Accordingly, behavioral health service consumers experience frequent crises, relapse, or other emergencies related to their conditions and need skills to manage and maintain recovery for improved physical and mental wellness (10). The President's New Freedom Commission on Mental Health stated that recovery is possible and is increasingly realized among behavioral health service consumers (11). It is critical that communities understand behavioral health need in terms of prevalence of conditions, access to care, and quality of mental health services.

Thomas R. Insel, former director of the National Institute of Mental Health (NIMH), stated that “data on prevalence, treatment, and mortality indicate that mental illness remains an urgent, unmet public health concern” (10). The most recent national data for behavioral health prevalence were collected in the early 2000's with the Collaborative Psychiatric Epidemiologic Surveys (CPES) (12, 13). Most communities lack the funding and/or capacity to conduct high quality behavioral health needs assessments. The absence of current prevalence makes estimating and planning how to prioritize local needs extremely difficult for local municipalities. Absent costly, time consuming “shoe leather” epidemiology that larger psychiatric epidemiologic studies employed, such as the CPES and Epidemiologic Catchment Area studies (14–17), local municipalities need a way forward to understanding mental health needs in their communities.

Local behavioral health agencies and organizations depend on data to marshal the limited resources available to address the behavioral health needs of their cities. Additionally, national estimates are reliant on state and local reporting cases of behavioral health. If there are problems with estimating needs and capacity at the local level, these discrepancies will aggregate up to the national level. Thusly, it is difficult to develop appropriate, proactive strategies to address behavioral health needs at the local or national levels. Needs assessments at the local level will likely have to rely on publically available data made available through city and state agencies as well as examining local service utilization and gaps in services.

The “patchwork” nature of mental health services makes it difficult to identify the types and adequacy of on-the-ground mental health services (18–20). Prevalence of behavioral health conditions are underestimated (3). Prevalence estimates are imperfect measures of need as most assessments do not include institutionalized individuals, such as those in psychiatric hospitals or prisons (12, 21). Additionally, treatment and epidemiologic data on the behavioral mental health of racial/ethnic minorities is limited (12, 22).

It is also critical to analyze the quality of behavioral services that are provided in community settings. Evaluation data indicate that the implementation of best practices and evidence-based treatment approaches, such as the use of evidence-based treatment techniques and intervention fidelity, is uneven in community settings (23–25). There are significant barriers to adopting and implementing best practices in community settings, particularly those that serve highly vulnerable populations which include but are not limited to training, organizational climate, and leadership (8, 26–28). Lack of access to and implementation of evidence-based practices compromises the quality of behavioral health care available at the community level (23, 29, 30). Another behavioral health care access issue is the availability of services, which is directly impacted by the number of providers available to provide care (22, 31, 32).

This needs assessment focused on the unique behavioral health needs of the city of St. Louis. Like many cities across the U.S., St. Louis has a behavioral health service system challenged by historic fragmentation, continuing shortages of behavioral health care, uneven and insufficient evidence-based care, and only emerging provision of integrated care. Additionally, the state of Missouri did not expand Medicaid in 2014 and has not subsequently expanded, even after a ballot initiative that passed in 2020 which would allow for more low-income Missourians to be served.

The context of St. Louis carries particular risk for the behavioral health of its residents, risks associated with racial segregation, income inequality, violence, and resource limited communities. St. Louis faces unique challenges, such as depopulation and race-related tension related to the shooting death of Michael Brown and subsequent protests in nearby Ferguson. St. Louis remains one of the most racially and ethnically segregated cities in the United States. The overall median household income is $36,809, which is substantially less compared to the median income for Missouri ($47,380) and St. Louis has a high poverty rate (27%). The overall high school dropout rate is also high at almost 13%. The unemployment rate is estimated to be 9% overall but hovers around 16% for African Americans. Nearly 20% of the population is uninsured. African Americans comprise the largest racial group (49%) followed by White residents (45%). Although the proportion of Latino residents overall is low (3.6%), there has been an increase of nearly 60% in the Latino population since 2010. See Table 1 for an overview of St. Louis sociodemographic characteristics.

**Table 1:** Sociodemographic characteristics of St. Louis (2009–2013).

	**% (***n***) or M (SE)**
**Age (18+)**	79.1% (252,407)
Median age	34.2 years (±0.2)
**Gender**	
Men	48.3% (154,082)
Women	51.7% (164,873)
**Race**	
White	44.9% (143,308)
Black or African American	48.6% (154,888)
Asian	2.8% (8,929)
Two or more races	2.5% (8,092)
**Ethnicity**	
Hispanic or Latino (of any race)	3.60%
**Immigration Status**	
U.S. born	297,570
Foreign born	21,395
**Income (household median)**	34,582
**Poverty rate**	27.40%
**Employment Status**	
In labor force	65.4% (170,072)
Not in labor force	34.6% (89,893)
Unemployed %	9.4% (24,367)
**Housing**	
Home ownership %	44.60%
**Marital Status**	
Married	28.70%
Separated	3.50%
Divorced	12.10%
Widowed	6.40%
Never married	4.90%
**Insurance Type**	
Private health insurance	54.9% (172,485)
Public health insurance	34.9% (109,761)
No health insurance	18.2% (57,317)

The St. Louis city Mental Health Board (MHB) is a taxing authority that receives a tax appropriation from the Community Mental Health Fund and aims to serve adults with behavioral health conditions. In 2014, MHB reached almost 2,900 consumers through its investment of almost $4 million to serve adults in need of behavioral healthcare. The MHB contracted a team of researchers to conduct a community behavioral health needs assessment in 2015. This paper presents the methods and results from a mixed methods approach from a civic-academic partnership to estimating behavioral health needs as well as service capacity and quality of mental health services in the city of St. Louis. The primary focus of the needs assessment was to identify the most common behavioral health problems and their variation by demographic and geographical factors. Understanding needs—those met by services and those remaining unmet—and knowing who has those needs is an important foundation for service delivery (17). Given funding and personnel constraints, this paper provides an example of an approach that may be considered in similar municipal settings throughout the country.

Our mixed methods approach centered on the following objectives:

- Identify adult behavioral health needs and priority populations in St. Louis;
- Determine adequacy of current mental health services;
- Identify key gaps and challenges in delivery of quality care.

## Methods

A mixed method approach was implemented, using both quantitative and qualitative data. The team obtained data about socio-demographic characteristics, adult mental health and substance abuse indicators and service utilization estimates of adults in St. Louis using secondary data (i.e., data that were administrative or collected by a different state or national agency) included sources that were either publically available data or administrative data drawn from the BJC HealthCare System (BJC). Publically available sources are listed below, with more detailed information in Table 2. Detailed discussions of these data are below. Key informant interviews were used for the qualitative portion of this needs assessment.

**Table 2:** Publically available data sources.

US Census Bureau American Community Survey, 2009–2013
Substance Abuse and Mental Health Services Administration
HRSA/American Hospital Association, Annual Survey of Hospitals
Missouri Department of Mental Health
Missouri Department of Health and Senior Services
US Department of Health and Human Services
University of Wisconsin Population Health Institute. County Health Rakings, 2013
Collaborative Psychiatric Epidemiologic Surveys

Quantitative data, especially those concerning the quality and availability of behavioral health services, are limited. Because no representative epidemiologic data has focused directly on St. Louis since the early 1990's (16, 33), our team performed a thorough scan of the most recent, publically available data, and identified data subsets, where possible, to the city of St. Louis. We focused on sources of data that: (1) focused on adults only; (2) focused on city of St. Louis only; and (3) contained information on behavioral health (mental health and/or substance abuse). Behavioral health indicators and service utilization in St. Louis city amongst adults were drawn from five major sources including: Missouri Department of Mental Health, Missouri Department of Health and Senior Services, Missouri Information for Community Assessment (MICA), Substance Abuse and Mental Health Services Administration (SAMHSA), and the United States Department of Health and Human Services (21).

In addition to data that were publically available, our team obtained data from BJC Healthcare, the region's largest non-profit health care delivery organization, to gain additional insight into the mental health need in the city. The majority of Americans seek behavioral health services in primary care settings or while seeking treatment for physical health problems (34–36). Therefore, the team obtained and analyzed data pertaining to St. Louis city adult residents who received mental health diagnoses and treatment from the BJC to better understand behavioral health need. Data on behavioral health care within primary care and other medical settings provides a complement to data available from behavioral health specialty treatment facilities. We analyzed administrative data from BJC Healthcare to determine the mental health diagnoses and location of BJC Healthcare facilities serving St. Louis residents from 2010 to 2015. Analyses were restricted to patients aged 18 years or older who were primarily seeking treatment for mental health conditions. History of any mental health problem during the past 5 years was ascertained from electronic medical records defined by International Classification of Disease Version 9 (ICD-9) diagnosis codes and procedures, drug orders for psychiatric medications, and facility seen.

To achieve a more complete understanding of the behavioral health needs of adults in the city, key informant interviews were used to provide a system, and provider level perspective of the behavioral health needs. Mental Health Board provided the team with a list of 19 stakeholders, which included a range of administrators, supervisors, and service providers representing organizations responsible for behavioral health services, health care, state level policy, and public safety. The team worked with the MHB to identify a list of topics to inform the interviews. Questions were then refined and a qualitative interview guide was developed by the team and approved by the MHB. The semi-structured, open-ended interview guide consisted of 12 questions that addressed the following topics: (1) priority populations; (2) current service gaps and needs; (3) service quality; and (4) quality of life for adults with behavioral health needs.

The interview guide was pilot tested by the team and two trained, experienced doctoral students conducted the interviews. The students were provided additional training specific to the needs assessment and conducted mock interviews with other members of the research team. The recruitment of key stakeholders occurred in three phases: (1) The first contact was made via e-mail by the MHB detailing the needs assessment and the importance of the stakeholder's participation and insight; (2) The second contact was a follow-up e-mail asking stakeholders whether or not they would be available to participate in the study; (3) The third contact was phone calls made by two doctoral students to schedule interviews with stakeholders who expressed interest in participating in the needs assessment. The team requested face-to-face interviews and all were scheduled in-person aside from two that were held over the telephone in order to accommodate stakeholders' schedules. The team completed twelve, key informant interviews, each lasting approximately 60 min. The Institutional Review Board at Washington University in St. Louis approved this study. The Committee waived the requirement for written informed consent for participants in this study since it did not involve the collection of private information or the testing of an intervention, in accordance with the national legislation and institutional requirements.

The research team conducted qualitative analysis using a four-step approach and Dedoose software. First, the two researchers worked independently to analyze the transcripts. The team randomly selected one interview to independently code. The researchers then compared codes and came to consensus on codes and code meaning (37). The research team participated in consensus meetings to provide additional insight. The researchers then randomly assigned the two doctoral students six interviews for primary coding and another six interviews for secondary coding. Codes were subsequently categorized into broader themes that will be discussed in the following section. Quotes were extracted from the codes for each theme and then re-evaluated to ensure that they captured the meaning of the themes (38).

## Results

### What Are the Behavioral Health Needs in St. Louis and Who Receives Behavioral Health Services?

As depicted in Table 3, data drawn from the Missouri Department of Mental Health in 2013 indicated that there were 3,156 cases of mood disorders, 2,429 cases of psychotic disorder, 701 cases of impulse control disorder, 567 cases of anxiety disorder, and 274 cases of personality disorder among adult St. Louis residents. The most commonly occurring behavioral health condition in St. Louis was depression. Data from 2014 show that 21.8% of Missouri residents live with depressive disorders. Anxiety disorder was the third most common disorder in the city.

**Table 3:** Psychiatric treatment services, City of St. Louis, 2013.

**Estimates**	
Mood disorder	3,156
Psychotic disorder	2,429
Impulse control disorder	701
Anxiety disorder	567
Personality disorder	274

*Source: Missouri Department of Mental Health; http://dmh.mo.gov/docs/ada/countylinks/psychtreatment/e463.pdf*.

Missouri Information for Community Assessment data indicated that 17.4% of African American and 25.5% of White St. Louis residents suffered from depression in the past year. Missouri Information for Community Assessment data indicated that 12-month estimates of alcohol dependence were 9% of the city population, experienced by nearly 20% of African Americans and 19% of White Americans. Alcohol and drug abuse treatment data from the Missouri Department of Mental Health (DMH) indicated that heroin represents the substance use disorder most likely to be treated in the city. Among individuals suffering from alcohol or substance abuse related disorders, rates of co-occurring mental health problems such as anxiety, depression, and psychotic disorder, were high.

As indicated in Table 4, treatment data from BJC Healthcare included 9,950 behavioral health related visits to 11 BJC system hospitals by 4,333 city residents between 2010 and 2015. The median age was 43 years of age. Over half (58%) of visits were by women, compared to men (42%). Most visits were from African American patients (58%) followed by White patients (38%). Less than 1% of patients were Asian, Latino, or Native American (39).

**Table 4:** Visits to BJC Healthcare by City of St. Louis residents for Mental Health conditions, 2010–2015.

**Behavioral health condition**	**Percent**	**Frequency**
Depression	39.2	3,903
Dsythymia	3.7	372
Anxiety	18.4	1,834
Bipolar	35.4	3,519
Alcohol dependence	9.8	973
Drug abuse	1.35	134

*n = 9,950*.

The three most common conditions patients sought treatment for were depression, bipolar disorder, and anxiety. The highest proportion of visits for mental health treatment was depression (39%), with 3,903 visits for depression during the 5-year observation period. The second most represented mental health condition treated within BJC was bipolar disorder (35%) with 3,519 visits. Another 18% of patients sought treatment for anxiety. Alcohol abuse treatment made up nearly 10% of visits and 1.3% of visits were for drug abuse treatment. Sixty percent of depression were with African American patients compared to White patients (36%). For bipolar disorder visits, 58% were by African Americans compared to White residents (36%). African Americans made up 55% of the anxiety disorder visits compared to 41% of White residents. These racial/ethnic differences in treatment findings diverge from those found with MICA data as well as data from national psychiatric epidemiologic studies (40–42).

### Behavioral Health Services Access

Data related to the availability of behavioral health services in St. Louis were drawn from Health Resources and Services Administration (HRSA) data as well as stakeholder interviews. These data reveal four critical gaps (1) psychiatrists, (2) behavioral health professionals (e.g. social workers, case managers, and nurses); (3) drug addiction services; and (4) stable and supportive housing. HRSA data indicated that there were only 46 psychiatrists in the city of St. Louis, reflecting a ratio of 14.5 per 100,000 residents. While St. Louis' supply of psychiatrists falls in the mid-range of national data, there are two major teaching hospitals within the city, which indicates that many psychiatrists based in the St. Louis are not engaged in treatment.

Key informants also indicated that there was a significant shortage of psychiatrists in St. Louis, which contributes to consumers' inability to secure appropriate medications. One behavioral health leader stated:

*There are not enough psychiatrists*. *Until the shortage of psychiatry is addressed, it will be difficult for people to be seen in a timely manner*.

The psychiatry shortage affects consumers with insurance as well. Another participant, stated:

*I hear from people who are trying to get a private psychiatrist. They have great insurance but can't get an appointment for three months. That's not uncommon. That's people with insurance*.

Limited substance abuse services was identified as another major shortage area, especially in light of steep increases in addiction, particularly heroin and opioids. Key informants noted that there are far too many consumers who need medical detoxification services relative to the number of facilities that that provide these services.

The need for increased coordination of care to improve services emerged as another major theme related to the availability of behavioral health services. The coordination of resource referrals and collaboration was highlighted as a barrier to quality behavioral health care. Interviewees noted that due to the complexity and fragmentation of behavioral health services, when staff leave agencies, the contacts they made at other agencies and facilities are often lost as well. Informants highlighted the need for professionals who can serve as facilitators and navigators for a fragmented healthcare system. Interviewees described a need for case manager/patient advocates to help individuals navigate the system and physically accompany them. Without these types of professionals, such as social workers, the quality of behavioral healthcare can be negatively affected. For example, a key informant stated:

*A lot of people need case managers to help them navigate all of their needs. If agencies could just provide a human being to help these folks figure out the maze … to get into a system of care or a system of help*.

Behavioral health leaders often focused on the need for a case manager/patient advocate system to help individuals navigate the system and physically accompany them to appointments. Through increased standardization of delivery/referral systems, existing resources may better meet the needs of individuals with behavioral health needs in a more efficient way. Some stakeholders expressed frustration with time spent looking for and contacting resources. For example, one participant stated:

*Coordinating these resources and then making sure everybody's aware of them and having the resources report back what they had this month or this week so that you don't spend a lot of time trying to identify where the resources are that this patient could potentially get. You've got to spend ten hours on the phone trying to track down things and it's a lot of work that nobody really has time for*.

Linkages and coordination between acute and community levels of behavioral healthcare were consistently highlighted as a critical factor in the provision of high quality care.

In addition to the shortage of behavioral health professionals available in St. Louis, key informants indicated that accessibility of behavioral health services was a major barrier to care. The majority of behavioral health providers are located in one central corridor of the city. Predominantly Black and high-poverty neighborhoods were underserved and several respondents indicated that there were very limited numbers or a complete lack of behavioral health providers in these communities. Behavioral health leaders indicated that the availability of providers closely parallels the racial composition of the city's neighborhoods and the areas with the highest level of need, particularly due to high levels of poverty and community-level trauma were neighborhoods with the poorest access to providers. Due to limited public transportation within the region, accessibility to behavioral health care for many vulnerable people in the region is even more challenging.

### Priority Populations

The top three priority populations identified by key stakeholders were (1) individuals with co-occurring mental health and substance abuse disorders, (2) emerging adult/youth transitioning, and (3) homeless/unstably housed individuals with behavioral health problems (39).

Interview data indicated that individuals with co-occurring behavioral health and substance abuse disorders were the service population highest in priority. Moreover, individuals with both mental health and addiction problems were seen as vulnerable to involvement with the criminal justice system, in part because they are “*visible downtown”* and as at risk for becoming homeless. For example, one participant stated:

*I would say [right now] those with dual diagnoses, so with both substance abuse, whether its drugs or alcohol and major psychiatric illness, usually it's going to be bipolar and schizophrenia*.

It was difficult for respondents to discern whether behavioral health or substance abuse disorders occurred first as one stakeholder added:

*Co-occurring, those partnered with drug and alcohol addictions. I don't know if it was a mental health disorder or the drug addiction, but that's the population we're seeing most frequently and we don't see any changes with*.

Additionally, individuals with behavioral health disorders and chronic medical illnesses, such as diabetes or chronic heart conditions were identified as a high priority. Respondents described this group as one that often falls between the health-behavioral health divide. One key informant stated:

*The behavioral health community views them as a population that the health community serves and the health community views them as a population that the behavioral health community's serving. Therefore, they fall through the cracks. They look at them as separate populations when they're one*.

Multiple stakeholders emphasized the importance of attending to the needs of young people transitioning from child to adult behavioral health. Respondents specifically discussed the lack of services in assisting these individuals navigate the adult behavioral health system. For example, one stakeholder stated:

*[Definitely] the transition, we don't have great transition into the adult system so we are now seeing more and more kids with behavioral health issues and teenagers, and then once they turn 18 or 21, they're out of the pediatric side and they have no clue how to access the adult side, so we see a lot of them lost and struggle in their early twenties and mid-twenties and eventually they'll come to us because they're injured or they're going to and they need a medical stability evaluation*.

Another participant echoed this sentiment, stating:

*[That's the other thing] that I hear a lot about, and this is… young people that are transitioning in, supposed to transition into the adult system, that they have a system of care that they're involved in as a youth, and then all of a sudden those services are no longer available, they can't get into the adult behavioral system*.

Almost all key informants explicitly named safe, stable, and accessible housing as a gap in current services. Stakeholders reported that housing is a major priority as stable and secure housing for individuals with behavioral health needs has important effects on treatment and long-term outcomes. Participants consistently discussed the integral role that housing plays in treatment retention and favorable outcomes. For example, one key informant participant noted the following about housing:

*We've come to realize over the years that if we don't provide people with stable housing of some kind, that their treatment is episodic, it's not continuous and as a consequence, they do well for short periods of time… They drop out of treatment, and we see them in the emergency room…*

Another participant noted the following:

*Housing… one of the things we know about people who have our types of problems is that the longer you can keep a person engaged, the better the outcome is. Unfortunately, there's a lot of money that has been allocated for the front-end, short-term crisis, detox, that sort of thing. But there's not a lot of emphasis on keeping people engaged for long periods of time, to really have a healthcare home or behavioral healthcare home model where they have a safe, healthy place to stay while they're getting services, becoming stabilized, and that sort of thing along with wrap around services*.

Stakeholders identified the lack of standardized outcomes and measurement tools in use across the field as a challenge. In order to establish clear evidence-based practice tenets, there is a need to identify and track measures related primarily to the process of care or intervention, rather than functional and symptom outcomes experienced by service users. Key informants indicated that there is tremendous variability in outcome indicators, making it difficult to describe overall outcomes across the varied efforts throughout St. Louis.

“*I think we're still missing outcomes.”* As one stakeholder stated, “*We've failed to agree on a common measures set*. *Probably 10, 12, maybe 20 different measures are in use.”*

In addition to the variation in outcome indicators, there were mixed perspectives about the implementation of evidence-based practices. Most participants indicated that behavioral health services in St. Louis are current and reported that behavioral health providers provide evidence-based treatment programs. Respondents identified the influence of funding agencies, including the MHB that have mandated the delivery of evidence-based treatment. Specific evidence-based approaches included in SAMHSA's list of evidence-based behavioral health programs and treatments such as Assertive Community Treatment and Cognitive Behavioral Treatment. However, stakeholders also indicated that there was variation in application and implication of evidence-based practices. Factors associated with this variation included the type of agency and the type of training providers had. Across the board, participants agreed that funding is a key lever in increasing provision of evidence-based care, and that provider training is key to ensuring delivery of evidence-based care.

## Discussion

Issues related to behavioral health pose as some of St. Louis' most serious public health problems which have been exacerbated in the wake of a continuing pandemic which as disproportionately affected the health and economic status of already vulnerable communities. The city has high rates of depression, substance use disorders and psychosis in addition to high levels of anxiety and co-occurring disorders (39). Their high prevalence notwithstanding, depression, substance disorders, and psychosis remain undertreated due in part to the city's shortage of behavioral health professionals, long waitlists for services, fragmented systems of care, and variable quality of care. Deficiencies in community surveillance and health system screening leave many behavioral health needs undetected, under-reported, and untreated. Existing treatment data indicate important needs as well as gaps in services.

Geographically, the city's behavioral health services reflect the racial residential segregation that is deeply entrenched in region, with those in predominantly African American areas having markedly lower access to services (39). Individuals living with mental illness and addiction need long-term stable housing and better access to transportation, to ensure their ability to travel to jobs and needed services. Critical barriers to outpatient behavioral health observed included affordability (e.g., insurance coverage) and accessibility, particularly geographical proximity and transportation. These access barriers are perceived to contribute to high reliance on emergency department care. Overall quality of behavioral healthcare was compromised by long wait lists, high staff turnover, highly variable usage of outcome indicators, and delivery of evidence-based treatment as well as challenges related to the fragmentation of the behavioral health system.

In response to the challenges outlined above, key informants suggested several recommendations to improve services, particularly with regard to coordination and collaboration. The most common response in relation to improving behavioral healthcare in St. Louis was to employ more behavioral health providers in order to increase access to care and to reduce the burden on psychiatrists and primary care providers. Overall, respondents noted that behavioral healthcare in St. Louis needs to be timelier and evidence-based, with a greater focus on prevention, responsiveness to early-stage expression of disorders, and long-term recovery. Behavioral health leaders in St. Louis also stressed the importance of standardizing and incentivizing collaborative service and the establishment of consistent outcomes that can be systematically tracked. This would permit analysis of quality of care and tracking of progress related to the provision and quality of behavioral health care services.

Efforts to address social determinants of health such as improving transportation services and promotion of employment programs would assist addressing the behavioral health needs in St. Louis. Critically, behavioral health leaders underscored the importance of long-term, stable housing solutions for behavioral health consumers seeking recovery, especially those who are homeless or do not have stable housing. Without stable housing, it is incredibly difficult for behavioral health consumers to maintain commitment to recovery and adhere to treatment plans. Although housing is fundamental for recovery, there are numerous challenges in securing appropriate housing solutions for consumers that need them. These challenges include long waitlists as well as the expense associated with maintaining housing for homeless and unstably housed consumers. Stakeholders also highlighted the challenge of simultaneously working to provide permanent supportive housing while attempting to help consumers that need rapid, rehousing solutions, which is often a problem for homeless consumers.

While these data sources are not perfect, usage of multiple sources of data, including information gleaned from key informant interviews, helps to create a picture of need at the local level as well as the opportunities to improve access to and quality of behavioral health services.

These data, while drawn from one city, are reflective of similar cities throughout the U.S. There have been substantial, nationwide financial cuts to behavioral health services, and de-institutionalization (43, 44). Extrapolating from these findings, it is critical to improve the availability and quality of behavioral health services to people who reside in urban contexts. Researchers are working to improve the dissemination and implementation of evidence-based practice into agencies and organizations, which will help to improve behavioral health treatment outcomes. On the prevention end, specific efforts to address the social determinants of health, especially those related to housing, education, and employment, are needed to improve overall well-being and preclude the development of behavioral health problems. These concerns have been underscored during the Coronavirus pandemic, of which communities of color have been disproportionately impacted. Furthermore, there is a need for funding at multiple levels (e.g. nationally, regionally, locally) to improve the availability and quality of data related to behavioral healthcare.

## Data Availability Statement

The datasets generated for this study will not be made publicly available Some data are proprietary and/or have identifiable information.

## Author Contributions

DH lead the drafting of the manuscript and conducted some of the analyses. SM assisted with the drafting of the manuscript and assisted in data collection. VM and LG led the analysis of qualitative data and drafting the methods section. EP assisted in all aspects of the project and drafting the manuscript. All authors contributed to the article and approved the submitted version.

## Conflict of Interest

The authors declare that the research was conducted in the absence of any commercial or financial relationships that could be construed as a potential conflict of interest.

## Publisher's Note

All claims expressed in this article are solely those of the authors and do not necessarily represent those of their affiliated organizations, or those of the publisher, the editors and the reviewers. Any product that may be evaluated in this article, or claim that may be made by its manufacturer, is not guaranteed or endorsed by the publisher.

## References

[1] Prince M Patel V Saxena S Maj M Maselko J Phillips MR. No health without mental health. Lancet. (2007) 370:859–77. 10.1016/S0140-6736(07)61238-0 17804063

[2] World Health Organization. The World Health Report 2001-Mental Health: New Understanding, New Hope. (2001). Available online at: http://www.who.int/whr/2001/en/whr01_en.pdf (accessed July 24, 2019).

[3] The WHO World Mental Health Survey Consortium. Prevalence, severity, and unmet need for treatment of mental disorders in the World Health Organization World Mental Health surveys. JAMA. (2004). 291:2581–90. 10.1001/jama.291.21.2581 15173149

[4] Brody DJ Pratt LA Hughes J. Prevalence of Depression Among Adults Aged 20 and Over: United States, 2013–2016. NCHS Data Brief, No 303. Hyattsville, MD: National Center for Health Statistics (2018). 29638213

[5] Alegría M Wong Y Mulvaney-Day N Nillni A Proctor E Nickel M. Community-based partnered research: new directions in mental health services research. Ethnic Dis. (2011). 21:S1–8-16. 22352075 PMC3653438

[6] Alegria M Chatterji P Wells K Cao Z Chen C Takeuchi D. Disparity in depression treatment among racial and ethnic minority populations in the United States. Psychiatr Serv. (2008) 59:1264–72. 10.1176/ps.2008.59.11.1264 18971402 PMC2668139

[7] Proctor E Silmere H Raghavan R Hovmand P Aarons G Bunger A. Outcomes for implementation research: conceptual distinctions, measurement challenges, and research agenda. Adm Policy Ment Health. (2011) 38:65–76. 10.1007/s10488-010-0319-7 20957426 PMC3068522

[8] Proctor EK Landsverk J Aarons G Chambers D Glisson C Mittman B. Implementation research in mental health services: an emerging science with conceptual, methodological, and training challenges. Adm Policy Ment Health. (2009) 36:24–34. 10.1007/s10488-008-0197-4 19104929 PMC3808121

[9] Kessler RC Berglund P Demler O Jin R Merikangas KR Walters EE. Lifetime prevalence and age-of-onset distributions of DSM-IV disorders in the national comorbidity survey replication. Arch Gen Psychiatry. (2005) 62:593–602. 10.1001/archpsyc.62.6.593 15939837

[10] Insel TR. Translating scientific opportunity into public health impact: a strategic plan for research on mental illness. Arch Gen Psychiatry. (2009) 66:128–33. 10.1001/archgenpsychiatry.2008.540 19188534

[11] New Freedom Commission on Mental Health. Achieving the Promise: Transforming Mental Health Care in America. Final Report. DHHS Pub. No. SMA-03-3832. Rockville, MD (2003).

[12] Jackson JS Torres M Caldwell CH Neighbors HW Nesse RM Taylor RJ. The National Survey of American Life: a study of racial, ethnic and cultural influences on mental disorders and mental health. Int J Methods Psychiatr Res. (2004) 13:196–207. 10.1002/mpr.177 15719528 PMC6878295

[13] Kessler RC Berglund P Chiu WT Demler O Heeringa S Hiripi E. The US National Comorbidity Survey Replication (NCS-R): design and field procedures. Int J Methods Psychiatr Res. (2004) 13:69–92. 10.1002/mpr.167 15297905 PMC6878537

[14] Cottler LB Hu H Smallwood BA Anthony JC Wu L-T Eaton WW. Nonmedical opioid pain relievers and all-cause mortality: a 27-year follow-up from the epidemiologic catchment area study. Am J Public Health. (2015) 106:509–16. 10.2105/AJPH.2015.302961 26691106 PMC4815712

[15] Gonzalez HM Croghan T West B Williams D Nesse R Tarraf W. Antidepressant use in black and white populations in the United States. Psychiatr Serv. (2008) 59:1131–8. 10.1176/ps.2008.59.10.1131 18832498 PMC2582403

[16] Regier DA Narrow WE Rae DS. The epidemiology of anxiety disorders: the epidemiologic catchment area (ECA) experience. J Psychiatr Res. (1990) 24:3–14. 10.1016/0022-3956(90)90031-K 2280373

[17] Robins L Regier D editors. Psychiatric Disorders in America: The Epidemiologic Catchment Area Study. New York, NY: The Free Press (1991).

[18] Maguire A French D O'Reilly D. Residential segregation, dividing walls and mental health: a population-based record linkage study. J Epidemiol Commun Health. (2016) 70:845–54. 10.1136/jech-2015-206888 26858342 PMC5013154

[19] Rapp CA Etzel-Wise D Marty D Coffman M Carlson L Asher D. Barriers to evidence-based practice implementation: results of a qualitative study. Community Ment Health J. (2010) 46:112–8. 10.1007/s10597-009-9238-z 19685185

[20] Unützer J Schoenbaum M Druss BG Katon WJ. Transforming mental health care at the interface with general medicine: report for the presidents commission. Psychiatr Serv. (2006) 57:37–47. 10.1176/appi.ps.57.1.37 16399961

[21] United States Department of Health and Human Services. Mental Health: Culture, Race, and Ethnicity: A Supplement to Mental Health: A Report of the Surgeon General. Rockville, MD: U.S. Department of Health and Human Services, Substance Abuse and Mental Health Services Administration, Center for Mental Health Services (2001). 20669516

[22] Alegria M Atkins M Farmer E Slaton E Stelk W. One size does not fit all: taking diversity, culture and context seriously. Adm Policy Ment Health. (2010) 37:48–60. 10.1007/s10488-010-0283-2 20165910 PMC2874609

[23] Beidas RS Aarons G Barg F Evans A Hadley T Hoagwood K. Policy to implementation: evidence-based practice in community mental health–study protocol. Implement Sci. (2013) 8:38. 10.1186/1748-5908-8-38 23522556 PMC3618103

[24] Insel TR Fenton WS. Psychiatric epidemiology: it's not just about counting anymore. Arch Gen Psychiatry. (2005) 62:590–2. 10.1001/archpsyc.62.6.590 15939836 PMC1586102

[25] Williams NJ Beidas RS. Annual Research Review: the state of implementation science in child psychology and psychiatry: a review and suggestions to advance the field. J Child Psychol Psychiatry. (2019) 60:430–50. 10.1111/jcpp.12960 30144077 PMC6389440

[26] Aarons GA. Mental health provider attitudes toward adoption of evidence-based practice: the Evidence-Based Practice Attitude Scale (EBPAS). Ment Health Serv Res. (2004) 6:61–74. 10.1023/B:MHSR.0000024351.12294.65 15224451 PMC1564126

[27] Aarons GA Palinkas LA. Implementation of evidence-based practice in child welfare: service provider perspectives. Adm Policy Ment Health. (2007) 34:411–9. 10.1007/s10488-007-0121-3 17410420

[28] Aarons GJ Horowitz JD Dlugosz LR Ehrhart MG. The role of organizational processes in dissemination and implementation research. In: Brownson RC Colditz GA Proctor EK editors. https://oxford.universitypressscholarship.com/view/10.1093/oso/9780190683214.001.0001/oso-9780190683214 *Dissemination* and Implementation Research in Health: Translating Science to Practice. New York, NY: Oxfored University Press (2012) p. 121–142. 10.1093/acprof:oso/9780199751877.003.0007

[29] Eiraldi R Wolk CB Locke J Beidas R. Clearing hurdles: the challenges of implementation of mental health evidence-based practices in under-resourced schools. Adv Sch Ment Health Promot. (2015) 8:124–45. 10.1080/1754730X.2015.1037848 26336512 PMC4553241

[30] Patterson Silver Wolf DA van den Berk-Clark C Williams S-L Dulmus CN. Are therapists likely to use a new empirically supported treatment if required? Journal of Social Work. (2017) 18:666–78. 10.1177/1468017317743138

[31] Cook BL Doksum T Chen CN Carle A Alegría M. The role of provider supply and organization in reducing racial/ethnic disparities in mental health care in the U.S. Soc Sci Med. (2013) 84:102–9. 10.1016/j.socscimed.2013.02.006 23466259 PMC3659418

[32] Cook LB Alegría M. Racial-ethnic disparities in substance abuse treatment: the role of criminal history and socioeconomic status. Psychiatr Serv. (2011) 62:1273–81. 10.1176/ps.62.11.pss6211_1273 22211205 PMC3665009

[33] Regier DA Myers JK Robbins LN Blazer DG Hough RL Eatton WW. The nimh epidemiologic catchment area program: historical context, major objectives, and study population characteristics. Arch Gen Psychiatry. (1984) 41:934–41. 10.1001/archpsyc.1984.01790210016003 6089692

[34] Gonzalez HM Vega WA Williams DR Tarraf W West BT Neighbors HW. Depression care in the United States: too little for too few. Arch Gen Psychiatry. (2010) 67:37–46. 10.1001/archgenpsychiatry.2009.168 20048221 PMC2887749

[35] U.S. Preventive Services Task Force. Screening for depression: recommendations and rationale. Ann Intern Med. (2002) 136:760–4. 10.7326/0003-4819-136-10-200205210-00012 12020145

[36] Stockdale SE Lagomasino IT Siddique J McGuire T Miranda J. Racial and ethnic disparities in detection and treatment of depression and anxiety among psychiatric and primary health care visits, 1995–2005. Med Care. (2008) 46:668–77. 10.1097/MLR.0b013e3181789496 18580385 PMC3956700

[37] Miles MB Huberman AM. Qualitative Data Analysis: An Expanded Sourcebook (2nd ed.). Sage Publications, Inc. (1994).

[38] Braun V Clarke V. Using thematic analysis in psychology. Qual Res Psychol. (2006) 3:77–101. 10.1191/1478088706qp063oa

[39] George Warren Brown School of Social Work. Saint Louis Mental Health Board Needs Assessment. St. Louis, MO: Washington University in St. Louis (2015).

[40] Jackson JS Knight KM Rafferty JA. Race and unhealthy behaviors: chronic stress, the HPA axis, and physical and mental health disparities over the life course. Am J Public Health. (2010) 100:933–9. 10.2105/AJPH.2008.143446 19846689 PMC2853611

[41] Mezuk B Abdou CM Hudson D Kershaw KN Rafferty JA Lee H. “White box” epidemiology and the social neuroscience of health behaviors: the environmental affordances model. Soc Ment Health. (2013) 3:10.1177/2156869313480892. 10.1177/2156869313480892 24224131 PMC3820104

[42] Mouzon DM. Relationships of choice: can friendships or fictive kinships explain the race paradox in mental health? Soc Sci Res. (2014) 44C:32–43. 10.1016/j.ssresearch.2013.10.007 24468432

[43] Medford-Davis LN Beall RC. The changing health policy environment and behavioral health services delivery. Psychiatr Clin North Am. (2017) 40:533–40. 10.1016/j.psc.2017.05.013 28800807

[44] Thornicroft G Deb T Henderson C. Community mental health care worldwide: current status and further developments. World Psychiatry. (2016) 15:276–86. 10.1002/wps.20349 27717265 PMC5032514

